# *Aedes aegypti* Saliva Alters Leukocyte Recruitment and Cytokine Signaling by Antigen-Presenting Cells during West Nile Virus Infection

**DOI:** 10.1371/journal.pone.0011704

**Published:** 2010-07-22

**Authors:** Bradley S. Schneider, Lynn Soong, Lark L. Coffey, Heather L. Stevenson, Charles E. McGee, Stephen Higgs

**Affiliations:** 1 Department of Pathology, Center for Biodefense & Emerging Infectious Diseases, University of Texas Medical Branch, Galveston, Texas, United States of America; 2 Department of Microbiology and Immunology, University of Texas Medical Branch, Galveston, Texas, United States of America; 3 Institut Pasteur, Department of Virology, Viral Populations and Pathogenesis Group, Paris, France; University of California Los Angeles, United States of America

## Abstract

West Nile virus (WNV) is transmitted during mosquito bloodfeeding. Consequently, the first vertebrate cells to contact WNV are cells in the skin, followed by those in the draining lymph node. Macrophages and dendritic cells are critical early responders in host defense against WNV infection, not just because of their role in orchestrating the immune response, but also because of their importance as sites of early peripheral viral replication. Antigen-presenting cell (APC) signals have a profound effect on host antiviral responses and disease severity. During transmission, WNV is intimately associated with mosquito saliva. Due to the ability of mosquito saliva to affect inflammation and immune responses, and the importance of understanding early events in WNV infection, we investigated whether mosquito saliva alters APC signaling during arbovirus infection, and if alterations in cell recruitment occur when WNV infection is initiated with mosquito saliva. Accordingly, experiments were performed with cultured dendritic cells and macrophages, flow cytometry was used to characterize infiltrating cell types in the skin and lymph nodes during early infection, and real-time RT-PCR was employed to evaluate virus and cytokine levels. Our *in vitro* results suggest that mosquito saliva significantly decreases the expression of interferon-β and inducible nitric oxide synthase in macrophages (by as much as 50 and 70%, respectively), whilst transiently enhancing interleukin-10 (IL-10) expression. *In vivo* results indicate that the predominate effect of mosquito feeding is to significantly reduce the recruitment of T cells, leading the inoculation site of mice exposed to WNV alone to have up to 2.8 fold more t cells as mice infected in the presence of mosquito saliva. These shifts in cell population are associated with significantly elevated IL-10 and WNV (up to 4.0 and 10 fold, respectively) in the skin and draining lymph nodes. These results suggest that mosquito saliva dysregulates APC antiviral signaling, and reveal a possible mechanism for the observed enhancement of WNV disease mediated by mosquito saliva via a reduction of T lymphocyte and antiviral activity at the inoculation site, an elevated abundance of susceptible cell types, and a concomitant increase in immunoregulatory activity of IL-10.

## Introduction

The skin and draining lymph nodes are the earliest sites of arbovirus infection. When the skin tissue is damaged during mosquito feeding, an assortment of soluble mediators and cells contribute to the response. Soluble mediators released by injured cells activate polymorphonuclear neutrophils and other types of cells that accumulate at the site of injury [1]. Concurrent with neutrophil accumulation, other cells migrate from both the epidermis [Langerhans cells (LC) and γδ T cells] and the blood (granulocytes, monocytes, natural killer cells, and CD4/CD8 T-cells). Resident, and later migratory, dendritic cells (DC) and macrophages also play an essential role. Thus, once the skin equilibrium is disrupted via injury or infection an inflammatory response is initiated characterized by the migration of varying waves of cells into the dermis. Following their recruitment, these cells play important roles in the early signaling that activates and orchestrates the immune response.

Both viral infection and mosquito saliva have the capacity to affect the migration of immune cells [2], [3], [4]. Previous research showed that cutaneous West Nile virus (WNV) infection leads to a decrease in LC density in the epidermis with a concomitant increase in LC concentration in draining lymph nodes [4]. Experiments with mice deficient in IL-1β revealed that IL-1β, but not tumor necrosis factor (TNF)-α, is required for this migration [2]. Neutrophils are among the first responders to mosquito feeding, and their homing to the site of blood-feeding is regulated by mast cell degranulation [3]. Elements of mosquito saliva can directly affect the cell types that migrate to the feeding site. The saliva of *Anopheles stephensi* contains a glycoprotein with an intense neutrophil chemotactic activity [5]. Moreover, *An. stephensi* saliva causes DC migration and directly triggers mast cell degranulation without a requirement for IgE [3]. Indirect effects of mosquito salivary anticoagulant proteins, such as *Aedes* anticoagulant-factor Xa [6], may down-modulate extravasation of some inflammatory cell types, and suppress complement pathways. It is possible that mosquito saliva could alter cell extravasation by affecting the mediators that trigger cell movement, as evidence from other hematophagous arthropods suggests [7], [8], [9], [10].

In addition to its effects on inflammatory cells, mosquito saliva also impacts T cell populations, increasing mortality and decreasing division rates [11]. Wasserman *et al.* [12] described inhibition of T- and B-cell proliferation in a dose-dependent manner concentrations as low as 0.15 salivary gland pairs/ml.

Antigen-presenting cells (APCs), such as macrophages and DCs facilitate both the destruction of invaders and initiation of pathogen recognition. The specific signals communicated by APCs, via soluble mediators, can have a profound effect on the vertebrate's immune response to the invader and the course of the infection. APCs are important in early arbovirus infection not just because of their role in orchestrating the immune response to the virus, but also as targets for early peripheral replication of virus. Indeed, with WNV infection, DCs are the primary site of viral replication [2]. The behavior of DCs and macrophages are fundamental to the pathogenesis of flaviviruses, although the balance of their beneficial versus deleterious effects depends on the regulation of their activity [13]. The inhibition of phagocytic activity or APCs results in enhanced viremia and neuroinvasion during WNV infection [14]. Some of the protective effects of macrophages appear to depend on the production of reactive oxygen intermediates, especially nitric oxide (NO) [15]. Despite the significant contributions of APCs to early suppression of flavivirus infection, it is likely that these cells play a duplicitous role, as they are also initial targets for WNV and Sindbis virus (SINV) after intradermal inoculation by mosquito vectors [2], [16].

The vigor of cell-mediated immunity is modulated by the abundance of key cytokines in the microenvironment surrounding the virus-infected APCs [17], [18]. APC-secreted cytokines play a key role in vertebrate defenses against viral infections via their effects on lymphocyte activation, proliferation, and differentiation, and also via their effects on inflammation and diapedesis, and through their anti-viral activity [19]. Expression patterns of cytokines dictate the specific response of a host to a pathogen, and early disruption of these proteins can have downstream repercussions that can affect the outcome of infection [20]. Thus the protective effects of macrophages and DCs are related to their ability to limit their own infection and to direct the antiviral immune response. Clearly, the early signaling activities are essential to successful defense against arbovirus infection and, consequently, alteration of their function may moderate disease outcome. The contribution of type I IFN in recovery from infection with arboviruses has been demonstrated *in vivo* by the therapeutic and prophylactic effects of administration of IFN-inducers or IFN [21], [22], [23]. Mice deficient in IFN-α receptor do not survive infection as compared to 70% survival in wild-type mice after a low-dose infection with Murray Valley encephalitis virus [24].

In the early stage of arbovirus pathogenesis, APCs are exposed to mosquito saliva, and thus saliva may impact the initial infection environment. Although there are currently no conclusive data to demonstrate that mosquito saliva directly effects APCs, based on studies with other hematophagous arthropods, such activity seems highly likely. For example, *Ixodes ricinus* salivary gland extract (SGE) inhibits the killing of *Borrelia afzelii* by macrophages, and reduces superoxide and NO production [25]. Saliva of the sand-fly *Phlebotomus papatasi* contains a potent inhibitor of macrophage protein phosphatase 1 and 2A [26], and reduces inducible NO synthase (iNOS) mRNA and NO production [26]. Sand fly saliva also inhibits antigen presentation [27] and TNF-α production, while enhancing IL-6, IL-10, and intracellular cyclic-AMP accumulation in macrophages [28]. The saliva of the biting midge, *Culicoides sonorensis*, was also shown to suppress NO secretion from peritoneal macrophages [29]. Our previous research demonstrates the potential for mosquito feeding to decrease the IFN response [30]. Additionally, *Aedes triseriatus* saliva has been shown to cause a reduced induction of IFN-α2 expression in L929 cells [31], while tick saliva can decrease expression of IFN-αβ in fibroblasts [32]

Considering that the saliva of related hematophagous arthropods have proven effects on APC function, and given the fact that early dysregulation of APC activities could alter virus pathogenesis, it is important to assess the effect that mosquito saliva has on APCs. Although independent studies have suggested that dermal cell migration can be effected by both mosquito feeding and virus inoculation (3–5), until now, studies to investigate the effects of mosquito saliva on cell migration during the initiation of an arbovirus infection have not been performed. The objective of this study was to investigate the influence of mosquito saliva on immune cell migration patterns in the dermis at the site of infection and in the draining lymph node following WNV infection. We also sought to determine if the presence of mosquito saliva during initial exposure of APCs to WNV alters the expression of important immune mediators. We hypothesized that mosquito saliva alters levels of murine macrophage, DC and T cell populations in the epidermis and draining lymph nodes during arbovirus infection, and modulates DC and macrophage signaling. Identifying the environments confronted by an arbovirus when transmitted in the presence or absence of saliva will provide an enhanced understanding of the early pathogenesis of naturally acquired infections.

## Materials and Methods

### Ethics Statement

The Institutional Animal Care and Use Committee at the University of Texas Medical Branch approved all animal protocols within this study. Accordingly this study was conducted adhering to both institutional and national guidelines for animal husbandry, and all possible steps were taken to minimize suffering in experiments.

### Virus

WNV strain 114 (GenBank accession numbers AY187013 and AY185907) [33] and SINV (strain AR339) were used for infections. Viruses were overlaid on cells at a multiplicity of infection (moi) of 1–5 diluted in PBS.

### Mice

Female, 4-week old C3H/HeJ mice were obtained from Harlan (Indianapolis, Indiana), and housed in a biosafety level-3 animal facility. Mice were divided into four groups (for each replicate 5 mice were utilized in per experimental group and 3 mice were used for the negative control group) and inoculated as follows: 1) PBS (negative control), 2) WNV alone, 3) mosquito feeding alone, and 4) WNV following the feeding of uninfected mosquitoes. Mice were acclimated to the animal facility for 1–2 weeks. Anesthetized mice were exposed to mosquito probing (restricted to the ear using a cardboard template surrounding a soft latex sleeve) for approximately 30 min when at least 10 mosquitoes had engorged. Following mosquito exposure (group 4), mice in groups 2 and 4 were inoculated intradermally in the pinna of the right ear with a standardized dose of WNV (10^4^ pfu in 10 µl of PBS). At 24 and 48 h post infection, mice in each group were sedated and euthanized. Tissues were processed for flow cytometry and cells from the skin were pooled. Mouse experiments were repeated three times.

### Mosquitoes and Mosquito Salivary Gland Extract (SGE)

*Aedes aegypti* mosquitoes were used for all experiments. Although Culex mosquitoes are very important for the natural avian cycle of WNV, the ornithophilic tendency of Culex species dictates that WNV may often rely on mosquitoes with more promiscuous feeding habits to infect vertebrates [34]. Anthropophilic mosquitoes, such as *Ae. aegypti*, are believed to serve as significant bridge vectors to humans [35], and thus act as a suitable model vector for our study focusing on the effect of mosquito feeding on WNV transmission to mammals. *Ae. aegypti* were reared and maintained in an insectary as previously described [36]. Female salivary glands were isolated [30] and resuspended in PBS. The solution was sonicated and centrifuged at 13,000 rpm for 10 min at 4°C to release salivary proteins and remove residual cellular debris. Unless noted otherwise, primary cells were treated with a concentration of 1 salivary gland pair/ml.

### Isolation of Resident Peritoneal Murine Macrophages

Macrophages were isolated from the unstimulated peritoneal cavity of 5 C3H/HeJ mice and pooled as previously described [29]. Resident peritoneal macrophages are advantageous since they are not activated and represent a homogeneous population. Macrophages were resuspended in Eagle's minimal essential medium supplemented with 2 mM glutamine, 15 mM HEPES buffer, 0.02% (w/v) sodium bicarbonate, 100 IU/ml penicillin, and 100 µg/ml streptomycin. Cells were plated overnight at 5×10^5^ per well in 24-well plates, and non-adherent cells were aspirated the following morning prior to treatment. Over 85% of the adherent cells were identified as macrophages by Diff-Quik staining [37]. Since the macrophages were derived from mice that are nonreactive to endotoxin, it is unlikely that results presented here are due to mosquito salivary gland contamination. Three independent replicates of this experiment were conducted.

### Isolation and Harvesting of Bone Marrow-Derived DCs

DCs were generated from pooled C3H/HeJ bone marrow (5 mice) using complete Iscove's modified Dulbecco's medium (IMDM, Invitrogen, Carlsbad, CA) containing 10% FBS and supplemented with 20 ng/ml of recombinant GM-CFS (eBioscience, San Diego, CA). Nonadherent DCs were harvested on day 8 and used in all experiments. Cells were enumerated and the yield of DCs was assessed by flow cytometry. Flow cytometry was performed as described previously, using antibodies specific for CD11c, MHC class II and CD86 [38]. Bone marrow DCs were plated in 500 µl of complete IMDM at a concentration of 5×10^5^ cells/well in a 24-well plate overnight prior to infection. Three independent replicates of this experiment were conducted.

### RNA Isolation

At 24 and 48 h post-infection, three wells of macrophages or DCs per group were sampled. Medium was removed from wells and replaced with 350 ml lysis buffer (Qiagen, Valencia, CA); Samples remained at −80°C until processing, when they were quickly brought to room temperature in a water bath and vortexed. RNA was extracted using Qiagen's RNAeasy kit following the manufacturer's protocol. Residual genomic DNA was removed from samples using TURBO DNA-free™ DNase treatment (Ambion, Austin, TX) and confirmed by performing real-time PCR on samples without reverse transcriptase.

### Cytokine Quantification

Cytokine expression in primary cells was determined by real-time RT-PCR. Primer-probe sets utilized include murine IL-4, IL-10, IL-12 p40, IFN-β, IFN-γ, GAPDH and WNV [30], [39]. The sequences (5′ to 3′) for the forward primer (f), reverse primer (r), and 6-carboxyfluorescein/black-hole quencher labeled probe (p) are as follows: GADPH – (f) TCACTGGCATGGCCTTCC, (r) TCTCCAGGCGGCACGT, (p) TTCCTACCCCCAATGTGTCCGTCGT; IFN-b – (f) CCATCATGAACAACAGGTGGAT, (r) GAGAGGGCTGTGGTGGAGAA, (p) CTCCACGCTGCGTTCCTGCTGTG; IFN-g – (f) TCAGCTGATCCTTTGGACCC, (r) TCTCAGAGCTAGGCCGCAG, (p) AGGAGAAGCCCAGAACTTCTGTCTCAAGTCAG; IL-12 (p40) – (f) TCAGTGTCCTGCCAGGAGG, (r) CAGTTCAATGGGCAGGGTCT, (p) TGTCACCTGCCCAACTGCCGAG; IL-4 – (f) TCATCGGCATTTTGAACGAG, (r) TTTGGCACATCCATCTCCG, (p) GCATGGCGTCCCTTCTCCTGTGA; IL-10 – (f) ACAGCCGGGAAGACAATAACTG, (r) CCGCAGCTCTAGGAGCATG, (p) ACCCACTTCCCAGTCGGCCAGAG; IL-2 – (f) CCTGAGCAGGATGGAGAATTACA, (r) TCCAGAACATGCCGCAGAG, (p) CCCAAGCAGGCCACAGAATTGAAAG; iNOS – (f) CAGCTGGGCTGTACAAACCTT, (r) CATTGGAAGTGAAGCGTTTCG, (p) CGGGCAGCCTGTGAGACCTTTGA; WNV – (f) CAGACCACGCTACGGCG, (r) CTAGGGCCGCGTGGG, (p) TCTGCGGAGAGTGCAGTCTGCGAT. To Amplify RNA, 2.5 µl of template RNA, 25 pmol FAM-labeled probe, 100 pmol of each primer, were mixed together in a total volume of 25 µl. Amplification conditions were: 2 min at 50°C, 10 min at 95°C followed by 15 sec at 95°C, and 1 min at 60°C for a total of 50 cycles. Taqman One-Step RT-PCR Master Mix (Applied Biosystems, Foster City, California) and standard Taqman protocols (Applied Biosystems) were employed for reactions [33]. Each experimental sample was tested in duplicate. The amount of template RNA for each cytokine gene was normalized to the constitutively-expressed housekeeping gene, GAPDH, tested in parallel in each assay. The level of cytokine mRNA was normalized to GAPDH to control for variations in RNA extraction, and reverse-transcriptase efficiency and is expressed as an average for each sample.

### Virus Titration

Cell supernatants were titrated as serial 10-fold dilutions on Vero cells as previously described [36]. Plates were incubated 37°C for seven days. The tissue culture infectious dose 50% endpoint titers were calculated by the method of Reed and Muench and are reported in log_10_TCID_50_ per milliliter [40].

### Preparation of single-cell suspensions from ear and lymph nodes

Mice were sedated and euthanized. Ears were surface sterilized with 70% ethanol, tape-stripped by repeated applications of cellophane tape to remove the stratum corneum, surgically removed with clean dissection scissors, and placed individually in the wells of a 24-well plate filled with 70% ethanol for 15 min. Draining lymph nodes of the sub-mandibular region were teased from surrounding fat and collected in 15 ml conical tubes on ice with IMEM. Preparation of single cell suspensions proceeded as described previously [33].

### Flow Cytometry Analysis

Cells were plated in 96-well V-bottom plates (10^6^ per well) and pelleted by a 3 min spin at 1,400 rpm and 4°C. Cells were treated with 100 µl of purified 2.4G2 antibody (1 µg/well) specific for murine FcγII/III receptors (Fc block, BD Biosciences) in FACS staining buffer for 20 min at 4°C. After a brief wash, cells were resuspended in 50 µl with the appropriate amount of fluorochrome-conjugated monoclonal antibody specific for cell-surface antigens. Multicolor staining was performed for 30 min at 4°C in the dark, followed by three wash steps. For intracellular staining, cells were thoroughly resuspended in 100 µl per well of Cytofix/Cytoperm solution (BD Biosciences) for 20 min at 4°C. Following fixation, cells were washed twice in 250 µl 1× BD Perm/Wash solution. The intracellular antigen was stained in 50 µl BD Perm/Wash solution for 30 min at 4°C in the dark. Cells were pelleted, washed twice, resuspended in ∼300 µl of FACS staining buffer and transferred to flow cytometry tubes for cytometric analysis [33]. The dermal and LN inflammatory cells were identified by characteristic size forward scatter (FSC) and granulosity side scatter (SSC) combined with two-color analysis. Briefly, the DCs were identified as large cells, MHC class II (25-9-17) bright and CD11c (clone HL3) positive, and LCs by CD11c and Langerin (CD207) positive. The macrophages were identified by CD11b (clone M1/70) positive and high-level expression of the surface marker F4/80 (clone 6F12). The neutrophils were identified as small cells, Ly-6G bright (RB6-8C5) and negative for F4/80 (or MHC class II). The CD4 and CD8 T cells were identified by their small size and by CD4 (clone GK1.5) or CD8a (clone 53-6.7)-specific staining as well as CD3 (clone 145-2C11) staining. All antibodies were used concurrently with corresponding isotype controls. Antibodies were from BD Pharmingen (San Diego, CA). Lymphocyte populations were gated based on forward and side-scatter parameters, 20,000 events were collected using a BD FACSCalibur (BD Immunocytometry Systems, San Jose, CA) flow cytometer with CellQuest software (Immunocytometry Systems), and data were analyzed using FlowJo (Tree Star Inc., Ashland, OR) flow-cytometric analysis software.

### Statistics

Differences in cytokine mRNA production as well as cell population numbers were determined by Mann-Whitney test; values of *p*<0.05 were considered significant.

## Results

### Effect of Mosquito Salivary Gland Extracts on WNV Infection Peritoneal Macrophages

**Figure 1:**
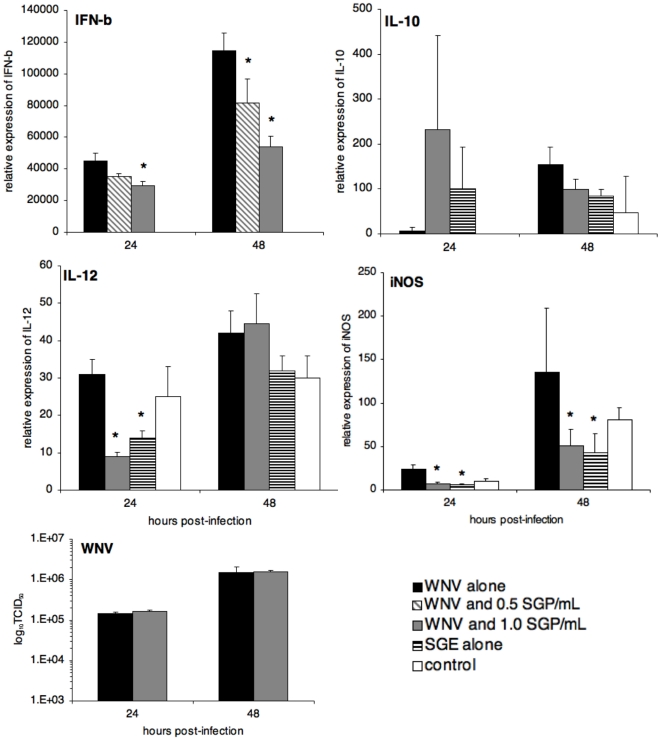
Cytokine expression and WNV titers in resident peritoneal macrophages following *in vitro* infection in the presence mosquito salivary gland extract (SGE). IFN-β, IL-10, IL-12, and iNOS levels were assessed by real-time RT-PCR at 24 and 48 h post-infection (MOI = 5). Their mRNA expression levels were normalized to those of the GAPDH gene. WNV production was quantified by titration on vero cells. The means ± standard deviation are shown, and an asterisk signifies a statistically significant difference (p<0.05) as compared to the group infected with WNV alone as determined by Mann-Whitney test. IFN-γ and IL-1β mRNA expression were found not to vary among groups (data not shown). Control groups were mock infected with PBS alone. Results were similar in three independent replicates.

To assess the effects of exposure to mosquito saliva on WNV infection, we examined changes in cytokine expression and virus titers at 24 h and 48 h post-infection (p.i.). WNV infection of macrophages stimulated type I IFN expression. Little to no IFN was detected in uninfected control macrophages or uninfected macrophages treated with SGE alone. At 24 h post- infection, macrophages treated with SGE (1.0 salivary gland pair (SGP)/ml) produced 33% less IFN-β mRNA than macrophages infected with WNV alone (Fig. 1; *p* = 0.038). At 48 h, the levels of IFN-β mRNA remained significantly lower in cells treated with SGE (*p* = 0.023). Also at 48 h p.i., there was a dose-dependent decline in IFN-β mRNA expression in macrophages treated with increasing amounts of SGE (0.5 to 1.0 SGP/ml). At the highest level of SGE (1.0 SGP/ml), IFN-β mRNA was reduced by >50% (*p* = 0.015) compared to mRNA levels in cells treated with WNV alone. In one of the four replicates, IFN-β expression levels were not significantly reduced, although it is possible that suboptimal culturing conditions accounted for this outlier given a higher than usual cellular mortality in this replicate. No difference in IFN-γ mRNA levels was detectable between groups in any of the replicates (data not shown). Expression of the pro-inflammatory cytokine IL-1β could not be detected in either WNV-infected or uninfected groups (data not shown).

At 24 h p.i., WNV alone led to minimal levels of IL-10 production by macrophages (Fig. 1). Treatment with SGE alone or in concert with WNV consistently resulted in elevated levels of IL-10 expression at 24 h. Although the difference was not statistically significant (*p* = 0.07), the trend of enhancement was observed for all replicates of this experiment. At 48 h p.i. IL-10 expression levels were comparable in all groups. Although IL-10 mRNA levels in two of the four replicates at 48 h were lower in the group treated with SGE and WNV, relative to the group treated with WNV alone (Fig. 1), this difference was not statistically significant and only occurred in half of the replicates.

Expression of IL-12 in macrophages varied according to treatment. At 24 h p.i., macrophages treated with SGE had the lowest level of IL-12 mRNA (Fig. 1). Macrophages co-treated with WNV and SGE had the lowest levels of IL-12 expression (*p* = 0.011); 68% less than IL-12 expression in macrophages infected with WNV alone. By 48 h, there was no discernible difference in IL-12 mRNA levels between groups.

Inducible nitric oxide synthase (iNOS) expression was observed in all treated macrophage groups. The group infected with WNV alone demonstrated the highest level of iNOS expression (Fig. 1), while levels in the group co-exposed to WNV and SGE were indistinguishable from control groups. Expression of iNOS in the group treated with WNV and SGE was 30.4% that of expression levels in the group infected with WNV alone (*p* = 0.021). At 48 h post-inoculation, wells co-treated with SGE had lower levels of iNOS mRNA than the WNV alone group (*p* = 0.038; Fig. 1).

Viral titers in WNV infected macrophages increased from 24 h to 48 h p.i. from an average of 1.20×10^5^ (24 h) to 1.34×10^6^ TCID_50_ (48 h) (Fig. 1). Between WNV-infected groups, viral titers were not different; suggesting that mosquito saliva does not alter WNV output resulting from peritoneal macrophages.

### Effect of Mosquito Salivary Gland Extracts on Sindbis virus (SINV) Infection Peritoneal Macrophages

**Figure 2:**
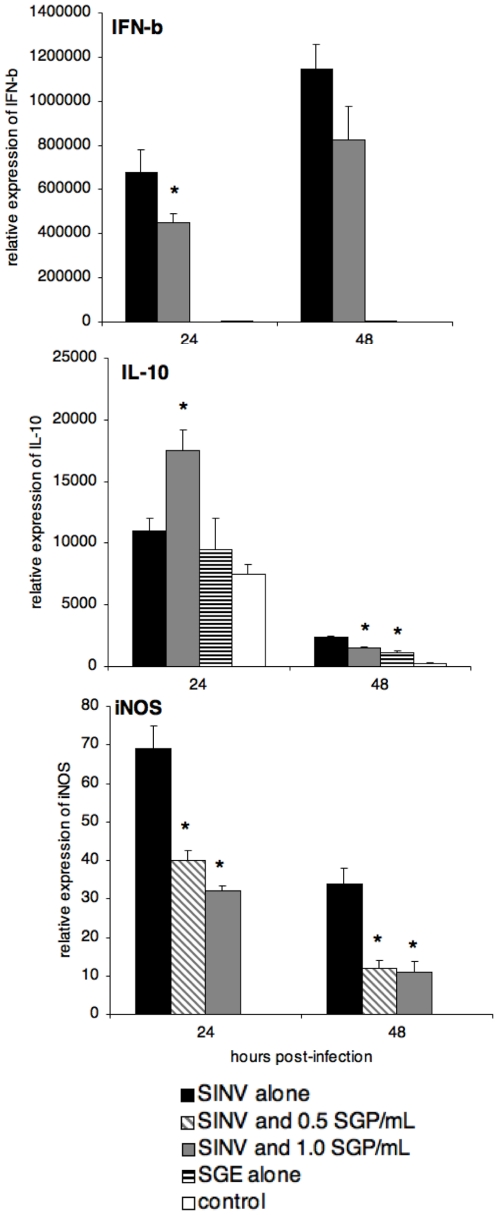
*In vitro* expression of cytokines in SINV-infected resident peritoneal macrophages. IFN-β, IL-10, and iNOS levels were assessed by real-time RT-PCR at 24 and 48 h post-infection (MOI  = 1). Their mRNA expression levels were normalized to those of the GAPDH gene. The means ± standard deviation are shown, and an asterisk signifies a statistically significant difference (p<0.05) as compared to the group infected with SINV alone as determined by Mann-Whitney test. IFN-γ, IL-12, and IL-1β mRNA expression, as well as SINV titers (assessed by titration on cell culture) were found not to vary among groups (data not shown). Control groups were mock infected with PBS alone. These results are representative of two independent replicates.

To determine if the effect of mosquito saliva on macrophages during arbovirus infection is virus-specific, the effect of exposure to mosquito saliva on macrophage function during SINV infection was also examined. Interferon expression was detected in all groups infected with SINV (Fig. 2). IFN-β mRNA levels were diminished by 40% at 24 h p.i. in groups exposed to SGE concurrent with SINV infection (*p* = 0.032). This trend was sustained through 48 h, but the suppression was only marginally significant (*p* = 0.052; Fig. 2). As with WNV infection of macrophages, the levels of IFN-γ mRNA did not vary significantly between groups at either time point (data not shown).

At 24 h p.i., SINV alone induced relatively low levels of IL-10 production by macr^6^ophages (Fig. 2). In contrast, exposure to SINV with SGE was associated with a 50% increase in mRNA levels of IL-10 (*p* = 0.024). By 48 h. IL-10 mRNA expression was detected in all groups of macrophages; levels in macrophages infected with SINV were elevated. Macrophages infected with SINV in the presence of SGE demonstrated significantly lower IL-10 expression levels than those infected with SINV only (Fig. 2, *p* = 0.045). Expression of IL-12 mRNA in macrophages was invariant across treatment groups at 24 h and 48 h p.i. (data not shown).

The iNOS expression was detected in all treatment groups except for uninfected controls. Macrophages infected with SINV alone had the highest level of iNOS expression at 24 h p.i. (Fig. 2). The levels of iNOS expression in macrophages exposed to SINV plus 0.5 or 1.0 SGP/ml were reduced by 42% and 49%, respectively (*p* = 0.012). Expression of iNOS at 48 h was also reduced by 65% in macrophages treated with SINV and SGE, compared to the group infected with SINV alone (Fig. 2; *p* = 0.018).

Viral titers increased from an average 4.6 log_10_ TCID_50_ at 24 h to 5.2 log_10_ TCID_50_ at 48 h p.i. (data not shown). Between SINV-infected groups, virus titers were equal, suggesting that, as with WNV, mosquito saliva administered *in vitro* does not alter SINV production in peritoneal macrophages.

### Effect of Mosquito SGE on Bone Marrow-Derived DCs

**Figure 3:**
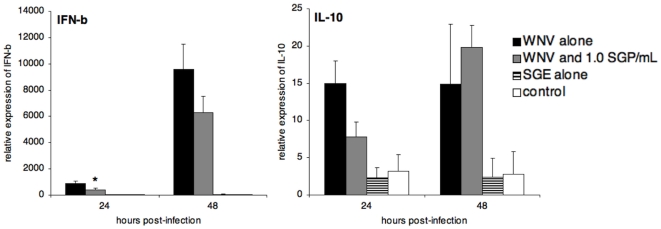
Cytokine mRNA expression in bone marrow-derived DCs following infection with WNV. IFN-β and IL-10 levels were assessed by real-time RT-PCR at 24 and 48 h post-infection (MOI = 5). Their mRNA expression levels were normalized to those of the GAPDH gene. The means ± standard deviation are shown, and an asterisk signifies a statistically significant difference (p<0.05) as compared to the group infected with WNV alone as determined by Mann-Whitney test. IFN-γ, IL-2, IL-4, IL-12, and IL-1β mRNA expression, as well as WNV titers (assessed by titration on cell culture) were found not to vary among groups (data not shown). Control groups were mock infected with PBS alone. This experiment was repeated three times.

To examine the effects of exposure to mosquito saliva on DC function during WNV infection, we measured cytokine expression in bone marrow-derived DCs at 24 h and 48 h p.i. WNV infection of DCs resulted in high type I IFN expression levels, with little to no IFN detectable in uninfected control DCs or DCs treated with SGE alone. At 24 h and 48 h p.i., WNV-infected DCs treated with SGE produced IFN-β mRNA levels that were lower (in 2 of 3 replicates, difference not statistically significant) than those produced in DCs treated with WNV alone (Fig. 3). Expression of IFN-γ was not detected in any group of DCs (data not shown). Expression of the T_H_2 cytokine IL-4 was statistically no different in WNV-infected and uninfected groups at both time points (data not shown). In contrast to observations in macrophages at 24 h p.i., infection of DCs with WNV alone resulted in a small increase in IL-10 mRNA levels, although this difference was not significant (Fig. 3, *p* = 0.09). Treatment with SGE or medium alone also resulted in low IL-10 mRNA expression. By 48 h. IL-10 mRNA expression levels were higher than levels at 24 h in infected groups (Fig. 3). Expression of IL-12 in DCs did not vary between WNV-infected groups at either 24 or 48 h p.i. (data not shown).

Viral titers from infected DCs increased from 24 h to 48 h p.i. from an average of approximately 1.88×10^5^ and 2.17×10^5^ to 2.24×10^6^ and 2.20×10^6^ TCID_50_, above titers are shown in log_10_TCID_50_, suggest log transforming for consistency respectively, for groups treated with WNV alone and WNV with SGE. Virus titers were no different between WNV-infected groups (data not shown).

### Cellular Infiltration into Primary Sites of WNV Replication

**Figure 4:**
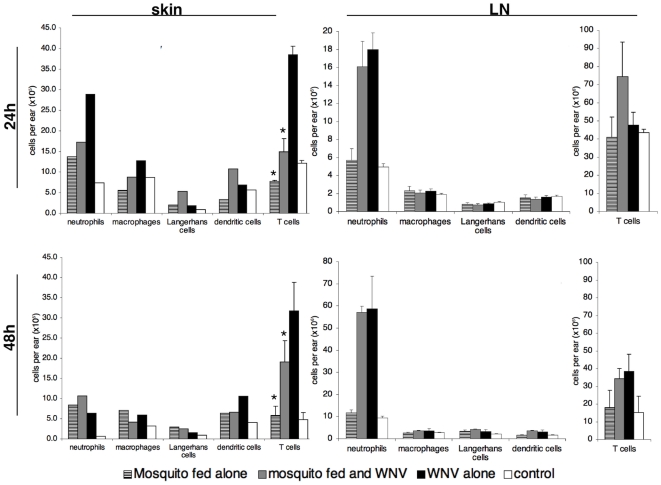
Leukocytes present in the dermal site of inoculation and the draining lymph nodes at 24 and 48 h after intradermal inoculation of WNV. Mice were inoculated with 10^4^ pfu of WNV in 10 µl of PBS with (n = 5) or without (n = 5) mosquito exposure, exposed to mosquitoes in the absence of WNV (n = 5), or mock infected with PBS alone (n = 3). Cells obtained from three ears per group were pooled, while lymph nodes were assessed individually. The populations of leukocytes were identified by staining and flow cytometry as described in Materials and Methods. The data shown for lymph nodes are the mean ± standard deviation (representative of 3 experimental replicates). The data for the skin are displayed as the cell numbers from the pooled samples, and the differences between experimental groups are representative of three independent replicates of this experiment. The skin T cells are represented as the mean ± standard deviation of three separate experiments; analysis of the other skin populations in this manner (across replicates) was not possible due to relatively large, yet proportional, differences that existed between replicates. Nonetheless, the differences between groups maintained a similar pattern in individual experiments, regardless of the variation in levels of particular cell types. An asterisk indicates values significantly different (p<0.05) than the values in the comparable cell populations of the group exposed to WNV alone as determined by Mann-Whitney test.

Components in saliva that alter hemostasis and inflammatory responses of the host may affect migration of resident-skin and blood-borne cells during early WNV infection. To investigate whether mosquito saliva modifies cellular content of the skin and draining lymph node, we evaluated the cellular responses to WNV inoculation and mosquito feeding. Compared to untreated animals, ear skin collected from mice in any of the exposure groups contained more neutrophils (Ly-6C/G positive cells; *p* = 0.039). At 24 h post-exposure, neutrophil levels were highest in mice inoculated with WNV with or without exposure to mosquito feeding (Fig. 4). By 48 h post-exposure, the level of neutrophils in skin increased to higher levels in the groups exposed to mosquitoes alone, mosquitoes and WNV, or WNV alone as compared to control tissue (Fig. 4), with 10- to 100-fold higher levels in skin exposed to these experimental conditions. Concurrently, ear tissues from mice that had been exposed to mosquito feeding (with or with out WNV inoculation) tended to have more neutrophils than those inoculated with WNV alone (Fig. 4).

In the skin, mosquito saliva exposure led to a significant reduction in T cells (CD3^+^ cells) (Fig. 4; *p* = 0.031). Interestingly, the skin of mice exposed to mosquito feeding alone contained significantly fewer T cells than even the skin of unexposed control mice (*p* = 0.038). Mice exposed to WNV alone had 2.8 and 1.6 times more skin inoculation site T cells than to mice exposed to mosquitoes and WNV inoculation at 24 (*p* = 0.014) and 48 h post-exposure (*p* = 0.031), respectively (Fig. 4). No corresponding decrease in T cells was observed in the draining lymph nodes (Fig. 4), although mice fed on by mosquitoes alone had slightly reduced levels of T cells, as compared to control mice at 24 h post exposure (*p* = 0.08).

At 24 h post-exposure, there was little change in the percent of the skin population made up of macrophages (F4/80 positive cells) in mice, other than a subtle increase in the group exposed to WNV alone as compared to controls (Fig. 4). At 48 h, the number of macrophages in the skin decreased slightly in WNV-exposed groups; levels were still higher than those in the control group (Fig. 4). Notably, at both 24 and 48 h post-exposure, mice that had been exposed to mosquito feeding prior to WNV inoculation tended to have fewer skin macrophages compared to mice inoculated with WNV alone. Although mosquito feeding increased levels of skin LCs only modestly, (Figs. 4) each replicate of the experiment found relatively higher levels of LCs in the inoculation site of mice exposed to mosquito feeding, and at 24 h post-exposure, LC numbers in the skin were elevated in the group of mice that were exposed to mosquitoes before WNV inoculation compared to WNV alone or control (Fig. 4). At 24 h, mice that were exposed to mosquito feeding and WNV tended to recruit DCs more rapidly in skin compared to WNV alone (observed in all 3 replicates), mosquitoes alone or control mouse skin; this trend was no longer apparent at 48 h post-exposure. Mice inoculated with WNV alone had a later influx of DCs into the skin.

### IL-10 expression and WNV replication in vivo

**Figure 5:**
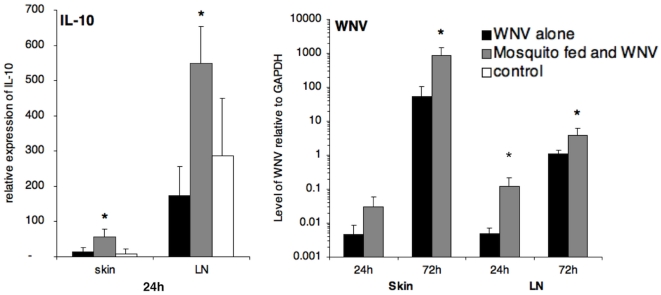
IL-10 and WNV RNA levels in the skin and draining lymph nodes following intradermal inoculation with 10^4^ pfu of WNV in 10 µl of PBS. RNA levels were assessed by real-time RT-PCR at 24 and 72 h post-infection and normalized to those of the GAPDH gene. The means ± standard deviation are shown, and an asterisk signifies a statistically significant difference (p<0.05) as compared to the group infected with WNV alone as determined by Mann-Whitney test. Control groups were mock infected with PBS alone. n = 5/group; displayed results are representative of two experimental replicates.

Since *in vitro* IL-10 levels varied among groups treated with WNV and SGE, a subset of skin and LN cells from mice were assayed for IL-10 and WNV mRNA expression levels (Fig. 5). Levels of WNV RNA increased from 24 to 72 h p.i. in both groups and in skin and LN. In both skin and LN at 24 and 72 h p.i. levels of WNV RNA were higher in groups of mice that had been exposed to mosquito feeding immediately prior to WNV infection compared to levels in mice infected with WNV alone, (skin: *p* = 0.031 at 72 h, LN: *p* = 0.022/*p* = 0.039 at 24 and 72 h). In agreement with *in vitro* findings, IL-10 expression in skin and lymph nodes was 4.0 and 3.1 times greater, respectively, (24 h, skin: *p* = 0.041; LN: *p* = 0.019) in the mosquito-exposed WNV-infected group compared to levels in mice inoculated with WNV alone. At 72 h post-exposure there was no difference in IL-10 expression between experimental groups (data not shown). Thus, overall, *in vivo* IL-10 data confirms our *in vitro* data with macrophages, showing a significant increase in mice exposed to mosquito saliva. Interestingly, in contrast to our cell culture data, *in vivo* WNV replication was consistently higher in groups exposed to mosquito saliva.

## Discussion

The aim of this study was to determine if mosquito saliva affects cell recruitment and/or the response of APCs to arbovirus infection, because such an alteration of the host response may help explain the mosquito-induced modulations previously reported in WNV pathogenesis studies [39], [41]. This question is of considerable significance given that macrophages and DCs are the primary sites of infection and the most peripheral components of a host immune response. Considering that mosquito saliva has powerful effects on immune cells [11], [12] and that the saliva of related hematophagous arthropods exerts suppressive effects on the microbicidal activity of APCs, it is particularly important to evaluate the effect of mosquito saliva on APCs during arbovirus exposure.

Previous studies have demonstrated that mosquito feeding and/or mosquito saliva can modulate the pathogenesis of arboviruses [42]. Although mosquito saliva has the potential to act on a multitude of host antimicrobial responses, we chose to assess a section of the response that is highly vulnerable to mosquito salivary factors that data from other hematophagous arthropods suggested was probable, namely, in the context of WNV transmission, the effect of the mosquito vector on cell recruitment and signaling. With regards to cell signaling, we found that during WNV infection mosquito feeding and mosquito saliva significantly altered iNOS, IFN-β, and IL-10 transcription levels. The observation that SINV-infected APCs also showed altered cytokine expression in the presence of mosquito saliva confirms that this effect is not unique to WNV infection.

Mosquito saliva consistently suppressed *in vitro* iNOS expression in APCs, especially macrophages. The catalytic activity of iNOS produces the reactive oxygen intermediate NO, which causes smooth muscle relaxation, inhibition of platelet activation, and induces direct and indirect immune responses [43]. The expression of iNOS is associated with a broad variety of transcription factors, including NF-κB, STAT-1α, IFN regulatory factor-1 (IRF-1), and IL-6 [44]. It is unclear how mosquito saliva suppresses iNOS expression in cells, but a number of regulatory mechanisms could be involved. For example, transforming growth factor β (TGF-β) and any factor that blocks L-arginine, the cofactor of iNOS, suppresses the production of NO in macrophages [44]. Additionally, T_H_2 cytokines IL-4 and IL-13 suppress iNOS transcription [44].

Mosquito saliva-induced suppression of iNOS could be a mechanism by which mosquito feeding exacerbates arbovirus disease, since some of the protective effects of macrophages appear to be mediated by NO production. For example, cells treated with an exogenous NO donor produced lower Japanese encephalitis virus titers [15]. This antiviral effect is attributed to the ability of NO to inhibit viral RNA synthesis, protein accumulation, and virus release from infected cells [15]. The inhibitory effect of NO on dengue virus infection is partly due to inhibition of RNA-dependent RNA polymerase activity, which down-regulates viral RNA synthesis [45]. NO also acts as an immune signaling molecule affecting differentiation, proliferation, and apoptosis of immune cells, cytokine production, and the expression of co-stimulatory and adhesion molecules [44]. NO also inhibits leukocyte adhesion to the endothelium and transmigration into tissue [46]. Due to the molecular nature of NO, its lack of defined receptors, its intra- and extra-cellular activity, and its pathogenic and beneficial effects during disease the effect of modulations in iNOS mRNA levels are hard to predict. Presumably, early in virus infection and in the periphery, where cells would come into contact with mosquito saliva, the effects of NO are more beneficial due to the antiviral activity within immune cells. Therefore a reduction in NO could be advantageous to the virus, by decreasing the ability of cells to control viral replication. Thus, a decrease in NO early in WNV infection could in part explain previously observations of enhancement of WNV disease after mosquito feeding [39].

Early studies on the effect of arthropod saliva on vertebrate cells demonstrated that mosquito saliva could reduce type I IFNs [30], [31], [32]. The present study shows that this effect of mosquito saliva occurs in APCs, early targets of arboviruses, during active WNV and SINV replication. A reduction in IFN-β expression is clearly an advantage to the virus, as type I IFNs comprise an important innate immune system control against viral infections [47], [48]. In general, IFN-β induces an antiviral state within cells through the up-regulation and activation of antiviral proteins and modulation of the adaptive immune response [47], and pretreatment of cells *in vitro* with IFN-α/β potently inhibits flaviviruses [49]. WNV nonstructural proteins inhibit IFN-α/β signaling by preventing JAK1 and Tyk2 phosphorylation and IFN-β gene transcription [50], therefore the antiviral effects mediated by IFN during flavivirus infection primarily benefit uninfected cells in the vicinity of infected cells. This suggests that a reduction of IFN production by cells exposed to virus, as observed in the presence of mosquito saliva, could enhance the susceptibility of nearby cells. If such an effect were to occur early during infection, when mosquito saliva is in proximity to infected cells, one would predict that the viability and spread of virus would be enhanced.

The early up-regulation of IL-10, as observed in arbovirus-infected macrophages in the presence of mosquito saliva, is also noteworthy. The enhancement of IL-10 in response to mosquito saliva confirms previous reports on arthropod saliva [30], [51], [52], [53], although this is the first time that it has been shown in macrophages or DCs. This cytokine is usually considered as immunoregulatory or immunosuppressive depending on the context, but research has identified profound effects of IL-10 expression early in infection [54]. Dengue virus-infected peripheral blood leukocytes produced mainly T_H_1 cytokines early after infection, but then shifted to producing IL-10 late after infection. [55]. Increased IL-10 production early in infection induces lasting T cell inactivation and decreases control of virus infection temporally associated with the establishment of effector T cell responses, even when high viral titers exist before the onset of T-cell activity. Moreover, *in vivo* antibody blocking of the IL-10 receptor completely prevented viral persistence when administered shortly after infection [56]. The interaction of immunosuppressive IL-10–producing APCs may be responsible for inducing T-cell unresponsiveness, which would allow for an enhancement of viral replication and blunted adaptive immune response. The significant elevation of IL-10 expression that we observed in the inoculation site and draining LNs in mice exposed to mosquito feeding immediately prior to WNV infection supports a central role for this cytokine in mediating the immunomodulatory effects of mosquito saliva during arbovirus infection. Our results are supported by a recent study which demonstrated that WNV infection is markedly diminished in IL-10 deficient mice, and pharmacologic blockade of IL-10 signaling increases survival of WNV-infected mice [57]. This suggests a significant proviral effect of IL-10 during WNV infection. Our *in vivo* study shows that mice exposed to mosquito saliva have consistently higher levels of WNV in the skin and draining LNs, and this is associated with an early enhancement of IL-10 expression. Even a transient enhancement of IL-10 expression by APCs, as observed here to be caused by *Ae. aegypti* saliva, could delay or suppress T cell activation, and thus impede initiation of the adaptive immune response, favoring the replication and spread of the virus. With a virus such as WNV, where specific virulence factors are not completely understood and alterations in primary viremia could turn an asymptomatic infection into encephalitis, even subtle changes brought about by mosquito saliva at peripheral sites cannot be ignored. Given its recently demonstrated [57] importance during WNV infection, the *in vivo* and *in vitro* elevation of IL-10 expression caused by mosquito saliva appears likely to be a key mediator of the averse effect of mosquito saliva on WNV infection. Taken as a whole, the enhancement of IL-10 expression, and reduction in iNOS and IFN-β expression, mediated by the inclusion of mosquito saliva during WNV infection, constitute a significant alternation in the early antiviral response.

In addition to the effect of mosquito saliva on APC signaling, mosquito feeding may also mediate its effect by altering cell populations at the feed site. Thus, we evaluated the role of mosquito saliva on cell density and type at the early sites of arbovirus replication, the inoculation site and draining lymph nodes. Neutrophils were among the first cells present after mosquito feeding, an observation that concurs with a previous study that examined the effects of mosquito feeding alone [3]. Demeure et al. [3] noted that *Anopheles stephensi* feeding alone leads to a migration of DCs to the draining LN, which peak at 48 h post exposure. Similarly, there was a tendency of higher levels of LCs observed here in groups of mice exposed to mosquito feeding (either with or without WNV inoculation). *Anopheles* mosquito feeding induces DC migration concurrent with stimulation by mast cell degranulation, and mast cell activation is followed by hyperplasia of the draining lymph node due to the accumulation of CD3^+^, B220^+^, CD11b^+^, and CD11c^+^ leukocytes [3]. Interestingly, mast cell degranulation caused by mosquito feeding appears to result in an immune-inhibitory environment, mediated by IL-10 [3]. WNV infection in the skin in the absence of mosquito feeding leads to DC migration to draining LNs [4] associated with an increase in MHC class II-expressing cells in the draining LN. Correspondingly, we observed an augmentation of MHC class II-expressing cells following WNV infection. Our data suggests that this migration occurs earlier in groups where mosquitoes fed immediately prior to virus inoculation. Byrne and associates [2] observed that 18 h after cutaneous WNV infection there is a significant increase in LC numbers in the draining LNs that corresponds to a decrease in epidermal LCs, as well as LN hyperplasia. Although our data demonstrate a subtle increase in skin LCs in the group exposed to mosquitoes, we did not observe a pattern of increase in the LN and decrease in skin LC counts. Nevertheless, our data shows a reduction trend of macrophage numbers in the skin following mosquito feeding and/or WNV infection corresponding to a rise in this cell population in the draining LN, suggesting, as would be expected, that these conditions cause macrophages to become activated and migrate to the LN. Overall, there is a trend towards more rapid recruitment of LCs and DCs in mice exposed to mosquito feeding in addition to WNV.

The observed decrease in T cells in WNV-infected skin after mosquito feeding supports previous studies, which showed that *Ae. aegypti* saliva significantly suppresses T cell activities at lower concentrations and enhances T cell death at higher concentrations [11], [12]. Notably, the current study demonstrates that this occurs during WNV infection. Wasserman and colleagues [12] observed that even very low concentrations of SGE caused an inhibition of T-lymphocyte proliferation. Dose-dependent suppression of cytokine secretion, particularly T_H_1 and proinflammatory cytokines, also resulted from SGE treatment of T cells [12]. Alterations in cytokine production by T cells could presumably alter cell influx. Both studies [11], [12] reported that *Ae. aegypti* SGE causes a significant decrease in T cell viability. The enhanced cell death, and subsequent decline in tissue level was more pronounced in CD4^+^ T cells. T cells provide an immediate response to peripherally inoculated WNV, limiting viral replication and invasion of the CNS in the first few days of infection [58]. Enhanced levels of T cells in the blood and recruitment to sites of viral infection have been documented in both human [59], [60] and murine [61] infections. The activity of T cells, is keenly important following cutaneous viral inoculation, and they are key in bridging the innate and adaptive immune response [62]. In principle, a reduction in CD4^+^ T cell numbers following mosquito feeding could influence WNV infection. A recent study with WNV [63], illustrated the important role that CD4^+^ T cells play; suppression or deficiency of this subpopulation during WNV infection in mice resulted in prolonged CNS infection and uniform lethality. Additionally, mice lacking CD4^+^ T cells had reduced IgG production and, later in infection, WNV-specific CD8^+^ T cell activation and trafficking to the CNS were compromised [63]. Mice that lack CD8^+^ T cells have higher CNS viral burdens and increased mortality rates after infection with WNV [64].

While no global shifts in the cell populations or migration are observed with the addition of mosquito feeding to the site of WNV inoculation, clear differences are evident. Most importantly is the reduction in T cells, although the earlier and relatively higher levels of APCs may play a role as well. This study expands our understanding of the early events subsequent to WNV infection, showing that mosquito feeding can affect the early inflammatory response to an arbovirus infection in a manner that favors the virus. Both the migration and signaling of APCs during WNV infection are altered in the presence of mosquito saliva. The recruitment of susceptible cell types is accelerated, while their expression of the immunosuppressive cytokine IL-10 is augmented and the expression of traditionally antiviral factors, IFNβ and iNOS, are reduced. The enhancement of IL-10 expression [30], [33], [39], [42], [51], [53] by mosquito saliva (particularly in *in vivo* experiments), should be given particular attention given the importance of this cytokine in early immune response orchestration. The consistent observation of reduced T-lymphocyte levels mediated by mosquito saliva [11], [12] also merits notice, as this perturbation may be key to the observed effect of mosquito saliva on arbovirus transmission and infection. Together, these *in vitro* and *in vivo* studies help define factors that contribute to WNV pathogenesis after natural infection and the role that the mosquito vector may play.

## References

[1] O'Flaherty JT, Cordes JF (1994). Human neutrophil degranulation responses to nucleotides.. Lab Invest.

[2] Byrne SN, Halliday GM, Johnston LJ, King NJ (2001). Interleukin-1beta but not tumor necrosis factor is involved in West Nile virus-induced Langerhans cell migration from the skin in C57BL/6 mice.. J Invest Dermatol.

[3] Demeure CE, Brahimi K, Hacini F, Marchand F, Peronet R (2005). Anopheles mosquito bites activate cutaneous mast cells leading to a local inflammatory response and lymph node hyperplasia.. J Immunol.

[4] Johnston LJ, Halliday GM, King NJ (2000). Langerhans cells migrate to local lymph nodes following cutaneous infection with an arbovirus.. J Invest Dermatol.

[5] Owhashi M, Harada M, Suguri S, Ohmae H, Ishii A (2001). The role of saliva of Anopheles stephensi in inflammatory response: identification of a high molecular weight neutrophil chemotactic factor.. Parasitol Res.

[6] Stark KR, James AA (1998). Isolation and characterization of the gene encoding a novel factor Xa-directed anticoagulant from the yellow fever mosquito, Aedes aegypti.. J Biol Chem.

[7] Anjili CO, Mbati PA, Mwangi RW, Githure JI, Olobo JO (1995). The chemotactic effect of Phlebotomus duboscqi (Diptera: Psychodidae) salivary gland lysates to murine monocytes.. Acta Trop.

[8] Montgomery RR, Lusitani D, De Boisfleury Chevance A, Malawista SE (2004). Tick saliva reduces adherence and area of human neutrophils.. Infect Immun.

[9] Macaluso KR, Wikel SK (2001). Dermacentor andersoni: effects of repeated infestations on lymphocyte proliferation, cytokine production, and adhesion-molecule expression by BALB/c mice.. Ann Trop Med Parasitol.

[10] Maxwell SS, Stoklasek TA, Dash Y, Macaluso KR, Wikel SK (2005). Tick modulation of the in-vitro expression of adhesion molecules by skin-derived endothelial cells.. Ann Trop Med Parasitol.

[11] Wanasen N, Nussenzveig RH, Champagne DE, Soong L, Higgs S (2004). Differential modulation of murine host immune response by salivary gland extracts from the mosquitoes Aedes aegypti and Culex quinquefasciatus.. Med Vet Entomol.

[12] Wasserman HA, Singh S, Champagne DE (2004). Saliva of the Yellow Fever mosquito, Aedes aegypti, modulates murine lymphocyte function.. Parasite Immunol.

[13] Chambers TJ, Diamond MS (2003). The Flaviviruses: Pathogenesis and Immunity; Chambers TJ, T.P. Monath, editor..

[14] Ben-Nathan D, Huitinga I, Lustig S, van Rooijen N, Kobiler D (1996). West Nile virus neuroinvasion and encephalitis induced by macrophage depletion in mice.. Arch Virol.

[15] Lin YL, Huang YL, Ma SH, Yeh CT, Chiou SY (1997). Inhibition of Japanese encephalitis virus infection by nitric oxide: antiviral effect of nitric oxide on RNA virus replication.. J Virol.

[16] Davis CW, Nguyen HY, Hanna SL, Sanchez MD, Doms RW (2006). West Nile virus discriminates between DC-SIGN and DC-SIGNR for cellular attachment and infection.. J Virol.

[17] Silva MC, Guerrero-Plata A, Gilfoy FD, Garofalo RP, Mason PW (2007). Differential activation of human monocyte-derived and plasmacytoid dendritic cells by West Nile virus generated in different host cells.. J Virol.

[18] Martina BE, Koraka P, van den Doel P, Rimmelzwaan GF, Haagmans BL (2008). DC-SIGN enhances infection of cells with glycosylated West Nile virus in vitro and virus replication in human dendritic cells induces production of IFN-alpha and TNF-alpha.. Virus Res.

[19] Thomson SA, Sherritt MA, Medveczky J, Elliott SL, Moss DJ (1998). Delivery of multiple CD8 cytotoxic T cell epitopes by DNA vaccination.. J Immunol.

[20] Pacsa AS, Agarwal R, Elbishbishi EA, Chaturvedi UC, Nagar R (2000). Role of interleukin-12 in patients with dengue hemorrhagic fever.. FEMS Immunol Med Microbiol.

[21] Vargin VV, Zschiesche W, Semenov BF (1977). Effects of tilorone hydrochloride on experimental flavivirus infections in mice.. Acta Virol.

[22] Taylor JL, Schoenherr C, Grossberg SE (1980). Protection against Japanese encephalitis virus in mice and hamsters by treatment with carboxymethylacridanone, a potent interferon inducer.. J Infect Dis.

[23] Haahr S (1971). The influence of Poly I:C on the course of infection in mice inoculated with West Nile virus.. Arch Gesamte Virusforsch.

[24] Lobigs M, Pavy M, Hall R (2003). Cross-protective and infection-enhancing immunity in mice vaccinated against flaviviruses belonging to the Japanese encephalitis virus serocomplex.. Vaccine.

[25] Kuthejlova M, Kopecky J, Stepanova G, Macela A (2001). Tick salivary gland extract inhibits killing of Borrelia afzelii spirochetes by mouse macrophages.. Infect Immun.

[26] Waitumbi J, Warburg A (1998). Phlebotomus papatasi saliva inhibits protein phosphatase activity and nitric oxide production by murine macrophages.. Infect Immun.

[27] Theodos CM, Titus RG (1993). Salivary gland material from the sand fly Lutzomyia longipalpis has an inhibitory effect on macrophage function in vitro.. Parasite Immunol.

[28] Soares MB, Titus RG, Shoemaker CB, David JR, Bozza M (1998). The vasoactive peptide maxadilan from sand fly saliva inhibits TNF-alpha and induces IL-6 by mouse macrophages through interaction with the pituitary adenylate cyclase-activating polypeptide (PACAP) receptor.. J Immunol.

[29] Bishop JV, Mejia JS, Perez de Leon AA, Tabachnick WJ, Titus RG (2006). Salivary gland extracts of culicoides sonorensis inhibit murine lymphocyte proliferation and no production by macrophages.. Am J Trop Med Hyg.

[30] Schneider BS, Soong L, Zeidner NS, Higgs S (2004). Aedes aegypti salivary gland extracts modulate anti-viral and TH1/TH2 cytokine responses to sindbis virus infection.. Viral Immunol.

[31] Limesand KH, Higgs S, Pearson LD, Beaty BJ (2003). Effect of mosquito salivary gland treatment on vesicular stomatitis New Jersey virus replication and interferon alpha/beta expression in vitro.. J Med Entomol.

[32] Hajnicka V, Kocakova P, Slovak M, Labuda M, Fuchsberger N (2000). Inhibition of the antiviral action of interferon by tick salivary gland extract.. Parasite Immunol.

[33] Schneider BS, McGee CE, Jordan JM, Stevenson HL, Soong L (2007). Prior exposure to uninfected mosquitoes enhances mortality in naturally-transmitted West Nile virus infection.. PLoS One.

[34] Wang LY (1975). Host preference of mosquito vectors of Japanese encephalitis.. Zhonghua Min Guo Wei Sheng Wu Xue Za Zhi.

[35] Turell MJ, Dohm DJ, Sardelis MR, Oguinn ML, Andreadis TG (2005). An update on the potential of north American mosquitoes (Diptera: Culicidae) to transmit West Nile Virus.. J Med Entomol.

[36] Higgs S, Schneider BS, Vanlandingham DL, Klingler KA, Gould EA (2005). Nonviremic transmission of West Nile virus.. Proc Natl Acad Sci U S A.

[37] Zhang X, Goncalves R, Mosser DM (2008). The isolation and characterization of murine macrophages.. Curr Protoc Immunol Chapter.

[38] Sanabria MX, Vargas-Inchaustegui DA, Xin L, Soong L (2008). Role of natural killer cells in modulating dendritic cell responses to Leishmania amazonensis infection.. Infect Immun.

[39] Schneider BS, Soong L, Girard YA, Campbell G, Mason P (2006). Potentiation of West Nile encephalitis by mosquito feeding.. Viral Immunol.

[40] McGee CE, Schneider BS, Girard YA, Vanlandingham DL, Higgs S (2007). Nonviremic transmission of West Nile virus: evaluation of the effects of space, time, and mosquito species.. Am J Trop Med Hyg.

[41] Styer LM, Bernard KA, Kramer LD (2006). Enhanced early West Nile virus infection in young chickens infected by mosquito bite: effect of viral dose.. Am J Trop Med Hyg.

[42] Schneider BS, Higgs S (2008). The enhancement of arbovirus transmission and disease by mosquito saliva is associated with modulation of the host immune response.. Trans R Soc Trop Med Hyg.

[43] Marletta MA (1994). Approaches toward selective inhibition of nitric oxide synthase.. J Med Chem.

[44] Bogdan C (2001). Nitric oxide and the immune response.. Nat Immunol.

[45] Takhampunya R, Padmanabhan R, Ubol S (2006). Antiviral action of nitric oxide on dengue virus type 2 replication.. J Gen Virol.

[46] Grisham MB, Granger DN, Lefer DJ (1998). Modulation of leukocyte-endothelial interactions by reactive metabolites of oxygen and nitrogen: relevance to ischemic heart disease.. Free Radic Biol Med.

[47] Pestka S, Krause CD, Walter MR (2004). Interferons, interferon-like cytokines, and their receptors.. Immunol Rev.

[48] Daffis S, Samuel MA, Suthar MS, Keller BC, Gale M (2008). Interferon regulatory factor IRF-7 induces the antiviral alpha interferon response and protects against lethal West Nile virus infection.. J Virol.

[49] Samuel MA, Diamond MS (2005). Alpha/beta interferon protects against lethal West Nile virus infection by restricting cellular tropism and enhancing neuronal survival.. J Virol.

[50] Guo JT, Hayashi J, Seeger C (2005). West Nile virus inhibits the signal transduction pathway of alpha interferon.. J Virol.

[51] Depinay N, Hacini F, Beghdadi W, Peronet R, Mecheri S (2006). Mast cell-dependent down-regulation of antigen-specific immune responses by mosquito bites.. J Immunol.

[52] Kopecky J, Kuthejlova M, Pechova J (1999). Salivary gland extract from Ixodes ricinus ticks inhibits production of interferon-gamma by the upregulation of interleukin-10.. Parasite Immunol.

[53] Zeidner NS, Higgs S, Happ CM, Beaty BJ, Miller BR (1999). Mosquito feeding modulates Th1 and Th2 cytokines in flavivirus susceptible mice: an effect mimicked by injection of sialokinins, but not demonstrated in flavivirus resistant mice.. Parasite Immunol.

[54] Brooks DG, Trifilo MJ, Edelmann KH, Teyton L, McGavern DB (2006). Interleukin-10 determines viral clearance or persistence in vivo.. Nat Med.

[55] Chaturvedi UC, Elbishbishi EA, Agarwal R, Raghupathy R, Nagar R (1999). Sequential production of cytokines by dengue virus-infected human peripheral blood leukocyte cultures.. J Med Virol.

[56] Ejrnaes M, Filippi CM, Martinic MM, Ling EM, Togher LM (2006). Resolution of a chronic viral infection after interleukin-10 receptor blockade.. J Exp Med.

[57] Bai F, Town T, Qian F, Wang P, Kamanaka M (2009). IL-10 signaling blockade controls murine West Nile virus infection.. PLoS Pathog.

[58] Wang T, Scully E, Yin Z, Kim JH, Wang S (2003). IFN-gamma-producing gamma delta T cells help control murine West Nile virus infection.. J Immunol.

[59] Agrati C, D'Offizi G, Narciso P, Abrignani S, Ippolito G (2001). Vdelta1 T lymphocytes expressing a Th1 phenotype are the major gammadelta T cell subset infiltrating the liver of HCV-infected persons.. Mol Med.

[60] Agrati C, D'Offizi G, Narciso P, Selva C, Pucillo LP (2001). Gammadelta T cell activation by chronic HIV infection may contribute to intrahepatic vdelta1 compartmentalization and hepatitis C virus disease progression independent of highly active antiretroviral therapy.. AIDS Res Hum Retroviruses.

[61] Rakasz E, Mueller A, Perlman S, Lynch RG (1999). Gammadelta T cell response induced by vaginal Herpes simplex 2 infection.. Immunol Lett.

[62] Wang T, Gao Y, Scully E, Davis CT, Anderson JF (2006). Gamma delta T cells facilitate adaptive immunity against West Nile virus infection in mice.. J Immunol.

[63] Sitati EM, Diamond MS (2006). CD4+ T Cell Responses are Required for Clearance of West Nile Virus from the Central Nervous System.. J Virol.

[64] Shrestha B, Diamond MS (2004). Role of CD8+ T cells in control of West Nile virus infection.. J Virol.

